# Individualized multimodal pain management for chronic perineal pain: A case report

**DOI:** 10.1097/MD.0000000000051175

**Published:** 2026-10-16

**Authors:** Xuemeng Ren, Lixin Cong

**Affiliations:** a Department of Pain Medicine, The Affiliated Hospital of Changchun University of Chinese Medicine, Changchun, Jilin, China.

**Keywords:** case report, pain, pain assessment, perineal pain

## Abstract

**Rationale:**

Persistent perineal pain is a challenging clinical condition that may substantially impair sleep, urination, daily activities, and psychological well-being. Accurate assessment and individualized multimodal management are essential for improving symptom control and patient participation in care.

**Patient concerns:**

A 68-year-old woman presented with chronic perineal pain and tenderness that had persisted for 3 years. The pain significantly interfered with her sleep, urination, and daily activities.

**Diagnoses:**

Clinical assessment suggested chronic perineal pain with neuropathic involvement of the pudendal nerve and lumbosacral or sacral nerve roots. The diagnosis was established based on the patient’s clinical manifestations, physical examination findings, and comprehensive evaluation.

**Interventions:**

The patient received a multimodal pain management approach, including oral pregabalin, computed tomography-guided sacral canal catheterization with targeted nerve-root adhesiolysis and ozone injection, continuous pain assessment, psychological support, pelvic floor muscle training, and individualized rehabilitation guidance.

**Outcomes:**

At the two-week follow-up, the visual analog scale score decreased from 8/10 to 3/10, and the pain no longer interfered with sleep. Approximately 3 weeks after discharge, the pain temporarily increased to 5/10 and subsequently stabilized at 4/10 at the one-month follow-up while the patient continued oral pregabalin.

**Lessons:**

Individualized multimodal pain management may effectively reduce pain intensity and improve sleep and daily functioning in patients with persistent perineal pain. Continuous assessment, patient participation, psychological support, and rehabilitation strategies are important components of comprehensive pain management.

## 1. Introduction

Perineal pain refers to idiopathic, insidious pain that occurs in the absence of identifiable anorectal or pelvic organ lesions.^[1]^ It is not a specific disease, but a clinical syndrome involving complex interactions among neurological, musculoskeletal, endocrine, psychological, and behavioral factors. Previous studies have suggested that perineal pain may involve nerve entrapment and neuropathic mechanisms, while persistent neuroinflammation and related signaling pathways may contribute to pain maintenance and associated psychological symptoms.^[2–7]^ Epidemiological studies conducted in Spain and the United States have reported prevalence rates of 18.7% and 9 to 16%, respectively.^[8,9]^

Pain is the predominant symptom of perineal pain and is often accompanied by urinary symptoms, with considerable clinical overlap between the 2.^[10]^ Management may include pharmacological therapy, physical rehabilitation, nerve blocks, image-guided procedures, and surgical or other interventional approaches, selected according to the suspected pain mechanism and the patient’s clinical condition.^[11,12]^ When conservative treatment does not provide adequate relief, interventional procedures may be considered. Previous studies have reported that neurolysis, peripheral nerve blocks, and ozone therapy may reduce pain associated with perineal neuropathy.^[13–16]^ Non-pharmacological interventions, including psychological support, rehabilitation guidance, and behavioral modification, are also important components of comprehensive management.

Severe pain is the most common complaint among patients with perineal pain and may substantially interfere with sleep, urination, mobility, and other daily activities.^[17]^ Chronic pain can reduce quality of life, increase the financial burden associated with repeated medical consultations, and contribute to psychological distress, which may further intensify pain and impair recovery. Accurate pain assessment and identification of factors associated with symptom persistence are therefore important for selecting appropriate interventions and adjusting care according to the patient’s response.

As a chronic condition, perineal pain requires not only diagnostic evaluation and symptom-directed treatment but also continuous and coordinated pain management.^[17]^ For pain management nurses, continuous assessment, patient education, psychological support, rehabilitation guidance, and identification of factors associated with symptom persistence are important for improving pain control and patient participation in care.^[18,19]^ However, most previous studies on perineal pain have focused primarily on diagnosis and medical treatment,^[20,21]^ while nursing strategies and continuity of pain management have received less attention. This case report describes the individualized multimodal management of an older patient with persistent perineal pain and highlights the role of nursing care in pain assessment, treatment participation, psychological support, rehabilitation, and post-discharge self-management.

## 2. Case report

### 2.1. Patient history

Patient Z, a 68-year-old female, presented with perineal pain, described as “sometimes like burning, sometimes like stabbing, and at other times pulsating with the rhythm of a heartbeat,” that had persisted for 3 years. The patient had undergone lumbar spine surgery 19 years previously and reported no other surgical history. She had reached menopause at 50 years of age and denied any history of pelvic inflammatory disease or other known gynecological disorders.

Prior to consultation at our hospital, she had previously attended another hospital, was diagnosed with “spinal nerve root inflammation,” and underwent acupuncture and physical therapy. However, as the pain recurred, she used various medications for symptom relief but felt no improvement. Pre-admission lumbar computed tomography showed degenerative changes of the lumbar spine and L5/S1 intervertebral disc herniation.

### 2.2. Examination data

Upon admission, the patient reported that the perineal pain was aggravated by standing, walking, and urination and was more severe at night. The pain disturbed her sleep and was accompanied by urinary frequency, urinary urgency, and a burning sensation during urination, which significantly affected her daily life. Urinalysis showed a leukocyte count of 87.3/μL, 15.7 leukocytes per high-power field, an epithelial cell count of 43.1/μL, and 7.758 epithelial cells per high-power field, indicating possible urinary tract inflammation. Other examinations revealed perineal tenderness and cutaneous hypersensitivity, with tenderness over the bilateral ischial spines. Physiological reflexes were present, no pathological reflexes were elicited, and her initial visual analogue scale (VAS) score was 8/10.

### 2.3. Clinical impression

The diagnosis of perineal pain was based on the patient’s three-year history of burning, stabbing, and pulsating pain localized to the perineal region, which was aggravated by standing, walking, and urination, together with perineal tenderness, cutaneous hypersensitivity, and tenderness over the bilateral ischial spines. These findings indicated a predominantly neuropathic pain pattern. Mild abnormalities on urinalysis were considered during the diagnostic assessment but were insufficient to explain the chronic pain characteristics and neurological examination findings. In addition, pre-admission lumbar computed tomography (CT) demonstrated lumbar degenerative changes and L5/S1 intervertebral disc herniation, suggesting possible lumbosacral or sacral nerve-root involvement.

In addition, the diagnostic process included assessment of the pain location, characteristics, duration, aggravating factors, associated symptoms, previous treatment history, neurological findings, perineal sensation, bilateral ischial-spine tenderness, urinalysis, and lumbar imaging. The Nantes criteria were also considered when evaluating possible pudendal nerve involvement.^[22]^ The perineal distribution of pain, burning and stabbing characteristics, and bilateral ischial-spine tenderness were compatible with pudendal neuralgia; however, the clinical findings also suggested possible sacral nerve-root involvement, and the symptoms were therefore not attributed exclusively to pudendal nerve entrapment. Based on the overall clinical findings, chronic perineal pain with suspected neuropathic involvement of the pudendal nerve and lumbosacral or sacral nerve roots was considered.

### 2.4. Intervention

Based on the patient’s preference and financial situation, a selective diagnostic pudendal nerve block was not performed, and thus the planned intervention was directed toward the suspected symptomatic sacral nerve-root region rather than the pudendal nerve alone. Considering the persistent neuropathic pain, bilateral ischial-spine tenderness, lumbar imaging findings, and insufficient response to previous conservative treatment, CT-guided sacral canal catheterization with targeted nerve-root adhesiolysis and drug administration was selected. CT guidance was used to confirm the catheter position and for the treatment to be delivered to the sacral plexus region corresponding to the patient’s symptoms.

On the first day of admission, the patient was prescribed pregabalin capsules 75 mg, twice daily (BID) for pain control. On the second day of admission, the patient underwent sacral canal catheterization for targeted nerve root adhesiolysis^[23]^ and ozone injection^[13]^ under CT guidance. After routine disinfection and local anesthesia, a puncture needle was inserted through the sacral hiatus, advanced perpendicular to the skin through the sacrococcygeal ligament until the periosteum was reached, then redirected cranially at an angle of approximately 15 to 30° to the skin and advanced approximately 2 to 3 cm into the sacral canal. The correct needle placement within the sacral canal was confirmed by perioperative CT. A continuous epidural catheter containing a guidewire was then advanced approximately 20 cm from the puncture site, and CT was used to confirm that the catheter tip was positioned in the anterior epidural space at the level of the sacral plexus corresponding to the symptomatic region. Next, targeted adhesiolysis was performed via the guidewire-containing catheter around the affected nerve root region. After removal of the guidewire, aspiration through the catheter confirmed the absence of blood and cerebrospinal fluid. A 26-mL mixture containing 5 mL lidocaine, 7 mg compound betamethasone injection, and 14 mL 0.9% sodium chloride solution was then administered through the catheter, followed by 10 mL ozone at a concentration of 30 μg/mL to further release epidural fibrous adhesions around the nerve roots. Then, the catheter was removed and the puncture site was covered with a sterile dressing. The procedure lasted approximately 30 min, and postoperatively, her VAS score decreased from 8/10 before treatment on March 26 to 6/10 after the procedure on March 27, remained at 6/10 on March 28, decreased to 5/10 by April 2, and further decreased to 3/10 at follow-up on April 16.

On Day 3, intravenous mannitol was administered BID to reduce postoperative nerve-root edema, while oral pregabalin was continued. Pain assessment on the same day showed partial relief, although the pain remained at a VAS score of 6/10. The patient expressed concern about pain recurrence, which further affected her sleep and pain perception. In addition to routine medication-related nursing care, the nurse provided emotional support and psychological counseling and guided the patient in pelvic floor muscle training, joint mobility exercises, relaxation, and breathing techniques. Family members were also encouraged to participate in supportive care, and the patient was informed of the importance of family understanding and support in pain management. With continued daily pain assessment, pain education, and rehabilitation guidance, the VAS score gradually decreased to 5/10.

### 2.5. Outcomes

On postoperative day 6, the patient reported a stable condition and requested discharge. She was provided with pain management instructions covering medication adherence, psychological support, physical rehabilitation, and the resumption of sexual activity after discharge. At the two-week follow-up, she reported continued adherence to the nursing recommendations and had learned to assess her pain independently. Her VAS score had decreased to 3/10, and the pain no longer interfered with sleep. At the one-month follow-up, the patient reported an overall improvement in her clinical symptoms, although the pain temporarily recurred approximately 3 weeks after discharge, with the VAS score increasing to 5/10 before stabilizing at 4/10. During the follow-up period, she continued oral pregabalin at 75 mg BID as instructed and received no additional pain intervention. The patient declined further follow-up at 3 months and was subsequently lost to follow-up. The clinical course and serial VAS scores are summarized in Table 1.

**Table 1:** Timeline of treatment, nursing interventions, and changes in VAS scores.

Date/time point	VAS score	Treatment, nursing intervention, or clinical outcome
March 26, 2024	8/10	Admission assessment; oral pregabalin 75 mg twice daily was initiated.
March 27, 2024	6/10	Sacral canal catheterization for targeted nerve root adhesiolysis and ozone injection under CT guidance; preoperative and postoperative education was provided.
March 28, 2024	6/10	Intravenous mannitol and oral pregabalin were continued. Psychological counseling, pelvic floor muscle training, joint mobility exercises, breathing exercises, and family education were provided.
April 2, 2024	5/10	The patient was discharged with instructions regarding medication adherence, psychological support, physical rehabilitation, and daily activities.
April 16, 2024	3/10	Two-week follow-up: pain intensity was reduced, and the pain no longer interfered with sleep.
Approximately 3 wk after discharge	5/10	The patient reported a temporary recurrence of perineal pain while continuing oral pregabalin 75 mg twice daily. No additional pain intervention was administered.
One month after discharge	4/10	The pain had stabilized at 4/10. The patient continued oral pregabalin 75 mg twice daily and reported an overall improvement in clinical symptoms.
Three months after discharge	Not available	The patient declined further long-term follow-up and was subsequently lost to follow-up.

CT = computed tomography, VAS = visual analogue scale.

### 2.6. Patient perspective

The patient reported that the persistent perineal pain had significantly affected her sleep, urination, and daily activities and that she was concerned about recurrence. She participated in treatment selection after the available options, expected benefits, potential risks, and costs were discussed with her and her family. At the two-week follow-up, she reported that the pain had decreased to 3/10 and no longer interfered with sleep. Approximately 3 weeks after discharge, the pain temporarily increased to 5/10, but subsequently stabilized at 4/10 while she continued oral pregabalin. At the one-month follow-up, she reported an overall improvement in her clinical symptoms compared with her condition before treatment.

### 2.7. Ethical considerations

This case report was reviewed and approved by the Ethics Committee of the Affiliated Hospital of Changchun University of Chinese Medicine on November 12, 2024 (Approval No. CCZYFYLL-SQ-2024-090). Written informed consent for participation and publication of the patient’s anonymized clinical information was obtained directly from the patient.

## 3. Discussion

Perineal pain is a complex clinical syndrome that may involve neurological, musculoskeletal, urological, gynecological, and psychological factors. In the present case, a comprehensive strategy combined pharmacological therapy, minimally invasive intervention, continuous pain assessment, psychological support, physical rehabilitation, and discharge management. This strategy was implemented, and the patient experienced short-term improvement in pain intensity, sleep, and daily functioning, indicating that coordinated pain management may provide clinical benefit when persistent perineal pain shows an inadequate response to previous treatment.

### 3.1. Pain assessment and individualized management

Accurate pain assessment forms the basis of treatment planning because pain remains a subjective experience influenced by biological, psychological, and social factors.^[24]^ The National Institute for Health and Care Excellence guideline for chronic pain emphasizes person-centered assessment that considers pain characteristics, associated symptoms, functional limitations, previous treatment experience, psychological status, and the patient’s priorities.^[25]^ For perineal pain, the assessment should also consider urinary and pelvic symptoms, possible neuropathic features, aggravating activities, and the effects of pain on sleep and intimate life.^[26,27]^

In this case, pain assessment was continuous, multidimensional, and quantitative. In addition to the VAS score, the nursing team assessed sleep, emotional responses, functional activity, treatment tolerance, and the patient’s understanding of symptom management, which allowed nursing interventions to be adjusted according to changes in her condition. Pain education also encouraged the patient to describe her symptoms accurately, understand the purpose of each intervention, and participate in pain self-assessment after discharge. We believe that such participation is important in chronic pain management because patients must continue medication, rehabilitation, and behavioral adjustment beyond the period of hospitalization.^[28]^

Psychological assessment was incorporated because persistent pain and anxiety may reinforce each other and increase sleep disturbance and pain perception.^[24]^ When the patient expressed concern about recurrence, emotional support, breathing exercises, relaxation training, and family involvement were added and complemented medical treatment by reducing psychological tension and improving her confidence in rehabilitation. Thus, the nursing approach focused not only on the numerical pain score but also on factors that could influence pain control and recovery.

### 3.2. Evidence and rationale for the selected interventions

The treatment strategy was selected according to the patient’s age, prolonged disease course, previous treatment response, lumbar imaging findings, severity of functional interference, psychological tolerance, financial circumstances, and personal preference. Pregabalin was used because the patient’s burning and stabbing pain, cutaneous hypersensitivity, and ischial-spine tenderness indicated a prominent neuropathic component, for which pharmacological treatment targeting neuropathic pain was clinically appropriate.^[29]^ The initial regimen of 75 mg BID allowed analgesic response and tolerability to be assessed in an older patient.

The minimally invasive intervention was selected because the patient had persistent pain despite previous therapies. The prescribed procedure combined catheter-based targeted nerve-root adhesiolysis, local anesthetic and anti-inflammatory medication, and ozone therapy under image guidance. Percutaneous epidural adhesiolysis is supported in selected patients with chronic spinal or radicular pain after an insufficient response to conservative management.^[23]^ In this case, the approach through the sacral hiatus enabled treatment of the suspected sacral nerve-root region and avoided direct perineal puncture, which was more acceptable to the patient. Ozone was included as an adjunct because of its proposed anti-inflammatory and analgesic effects. Clavo et al reported sustained pain improvement following ozone therapy in patients with refractory chronic pelvic pain secondary to cancer treatment.^[13]^ The evidence supporting ozone for perineal pain is less established than that for spinal or radicular pain; therefore, its use in this case was based on the clinical experience of the treatment team and the available evidence from related chronic pelvic and spinal pain conditions. The improvement observed after treatment supports the feasibility of this combined approach, although the respective contribution of ozone, adhesiolysis, epidural medication, pregabalin, and nursing interventions cannot be separated in a single case.

Pelvic floor muscle training was added to rehabilitation because pelvic floor dysfunction and increased muscle tension may contribute to chronic pelvic and perineal symptoms. Pelvic floor rehabilitation is commonly included in the management of vulvodynia, and recent evidence indicates that rehabilitative interventions may reduce pain and improve pelvic floor-related symptoms in affected women.^[30]^ A systematic review also found that pelvic floor physical therapy may improve pain and pelvic floor symptoms in patients with pelvic floor hypertonicity, vulvodynia, dyspareunia, and chronic pelvic pain.^[31]^ In the present case, pelvic floor training was combined with relaxation and breathing exercises and was adjusted according to the patient’s postoperative condition and tolerance. This approach made rehabilitation practical during hospitalization and suitable for continuation at home.

### 3.3. Treatment alternatives, safety, and shared decision-making

Alternative options included continued pharmacological treatment, further physical therapy, pudendal nerve block, ganglion impar block, and other targeted interventions. Pudendal nerve block may be considered when pudendal neuralgia is the predominant suspected mechanism and can provide both diagnostic and therapeutic information.^[20]^ In this case, the clinical findings suggested that the pain might involve a broader sacral nerve-root region rather than the pudendal nerve alone. Therefore, the selected procedure was directed toward the suspected nerve-root and sacral plexus region. Ganglion impar block has been used for pelvic and perineal pain, particularly when a sympathetic pain component is suspected and in patients with cancer-related pain.^[14,15]^ However, the patient’s previous lumbar surgery, imaging findings, and neuropathic presentation provided greater clinical support for the selected sacral approach. Additional physical therapy alone was also considered, but the patient had previously received acupuncture and physical therapy without sustained relief. Recent interventional and surgical literature also supports selecting procedures according to the suspected anatomical pain source, previous treatment response, imaging findings, and individual procedural tolerance.^[32]^

The advantages, disadvantages, and potential risks of the available treatments were discussed with the patient and her family before intervention. Shared decision-making was applied throughout the treatment process. The patient’s subjective experience, psychological tolerance, treatment expectations, and financial circumstances were considered together with the clinical findings. After receiving information regarding the available options, expected benefits, potential risks, and procedural characteristics, the patient selected the minimally invasive intervention because it avoided perineal puncture and was acceptable to her preferences. Shared decision-making is increasingly recognized as an important component of pain management because it supports communication, patient participation, and treatment selection based on both clinical evidence and individual priorities.^[33]^ Thus, our selected strategy involved one inpatient intervention followed by oral medication, pain education, and home-based rehabilitation to reduce repeated consultations and intermittent treatments while improving the patient’s ability to perform daily activities independently. The improvement in pain, sleep, and self-care also had the potential to reduce the immediate family caregiving burden. These observations represent the practical economic considerations of this case and should not be interpreted as formal evidence of cost-effectiveness.

### 3.4. Limitations

This case has several limitations that should be considered. First, a selective pudendal nerve block was not performed; therefore, the independent contribution of pudendal nerve involvement could not be determined. However, the selected intervention addressed the suspected sacral nerve-root region identified through the overall clinical and imaging assessment. Second, the treatment consisted of several concurrent components, and the clinical improvement cannot be attributed to a single intervention. Third, although the patient showed improvement during the available follow-up, the temporary increase in pain approximately 3 weeks after discharge indicates that symptom control was not completely stable. Finally, the patient declined further follow-up at 3 months, preventing evaluation of the longer-term outcome.

## 4. Conclusion

This case indicates that comprehensive pain management integrating continuous pain assessment, pregabalin, image-guided interventional treatment, psychological support, pelvic floor rehabilitation, and individualized discharge education may help relieve persistent perineal pain and improve sleep and daily functioning. The strategy was based on the patient’s clinical presentation, imaging findings, previous treatment response, psychological tolerance, financial circumstances, and personal preferences, with ongoing adjustment based on changes in pain and emotional status. Although the patient achieved improvements, the combined nature of the interventions and limited follow-up prevent determination of the independent effect of each treatment component or the long-term stability of symptom control. Further studies with standardized diagnostic assessment, longer follow-up, and clearly defined treatment pathways are required to evaluate the broader applicability of this comprehensive management approach.

## Author contributions

**Conceptualization:** Xuemeng Ren, Lixin Cong.

**Data curation:** Xuemeng Ren.

**Resources:** Xuemeng Ren.

**Writing – original draft:** Xuemeng Ren.

**Writing – review & editing:** Xuemeng Ren, Lixin Cong.
